# Cytomegalovirus chronic retinal necrosis with ganciclovir resistance: a case report

**DOI:** 10.1186/s12348-024-00434-w

**Published:** 2024-10-08

**Authors:** Julia Xia, Sanjana Kantipudi, Christopher C. Striebich, Andrés F. Henao-Martinez, Niranjan Manoharan, Alan G. Palestine, Amit K. Reddy

**Affiliations:** 1 Department of Ophthalmology, University of Colorado School of Medicine, 1675 Aurora Court, F731, Aurora, CO 80045 USA; 2 Division of Rheumatology, Department of Medicine, University of Colorado School of Medicine, Aurora, CO USA; 3 Division of Infectious Diseases, Department of Medicine, University of Colorado School of Medicine, Aurora, CO USA

**Keywords:** Cytomegalovirus, Chronic retinal necrosis, Viral retinitis, Ganciclovir, Letermovir, Leflunomide

## Abstract

**Background:**

Cytomegalovirus (CMV) chronic retinal necrosis (CRN) is a rare viral retinal infection that occurs in mildly immunocompromised people. It shares some features with both acute retinal necrosis and CMV retinitis. It is typically treated with combination intravitreal and systemic ganciclovir. We discuss the management of a case of CMV CRN with ganciclovir resistance.

**Case presentation:**

An 80-year-old female presented with one month of blurry vision in the left eye. She was being treated with abatacept, methotrexate, and prednisone for rheumatoid arthritis. Examination revealed anterior chamber and vitreous cell along with peripheral retinal whitening. Fluorescein angiogram showed diffuse retinal non-perfusion. Aqueous fluid PCR testing returned positive for CMV. The retinitis was initially controlled with oral and intravitreal ganciclovir, but then recurred and progressed despite these therapies. Ganciclovir resistance was suspected and the patient was switched to intravitreal foscarnet injections, along with oral letermovir and leflunomide, which lead to resolution of the retinitis. The patient has now continued with letermovir and leflunomide for approximately 2.5 years without reactivation of the retinitis or need for further intravitreal anti-viral injections and with adequate control of her rheumatoid arthritis.

**Conclusion:**

The incidence of CMV CRN may increase in the future as the use of non-cytotoxic immunosuppressive therapies that result in relatively mild immunosuppression also increases. Treatment with ganciclovir is effective but frequently leads to resistance, as in our case. In this situation, combination therapy with letermovir and leflunomide, particularly in the setting of rheumatoid arthritis where leflunomide can also have an anti-inflammatory effect, can be considered.

## Background

Cytomegalovirus (CMV) is a DNA Herpes family virus with human seroprevalence of up to 80% globally [1]. Systemic CMV infections in immunocompetent individuals are generally mild and self-limiting [2]. In severely immunocompromised states, such as in the setting of acquired immunodeficiency syndrome from human immunodeficiency virus (HIV), patients can develop a severe and vision-threatening retinitis from CMV. This typically presents with a slowly progressive granular retinal necrosis, retinal hemorrhages, and minimal intraocular inflammation [3]. With the widespread availability of highly-active antiretroviral therapy, the rates of CMV retinitis in patients with HIV have decreased dramatically [4, 5], and CMV retinitis is now more commonly seen following solid organ or hematopoietic stem cell transplant (HSCT) [6, 7]. 

More recently, patients with “milder” immunosuppression or immunocompromise arising, for example, from the use of systemic corticosteroids or non-cytotoxic therapies for systemic autoimmune disease, have been reported to develop a slowly progressive CMV-associated retinal necrosis that is associated with significantly more intraocular inflammation and neovascular complications than the previously characterized CMV retinitis. This phenotype was given the nomenclature “chronic retinal necrosis” (CRN) in a paper by Schneider et al. in 2013 [8]. Treatment for this disease has generally consisted of systemic ganciclovir, intravitreal ganciclovir or foscarnet, and the potential reduction or discontinuation of systemic immunosuppressive therapies [8–14]. 

While the topic of ganciclovir-resistant CMV has been extensively discussed in the context of CMV retinitis [6], no prior reports have discussed the management of ganciclovir resistance with CMV CRN. We describe such a case here.

## Case presentation

**Fig. 1:**
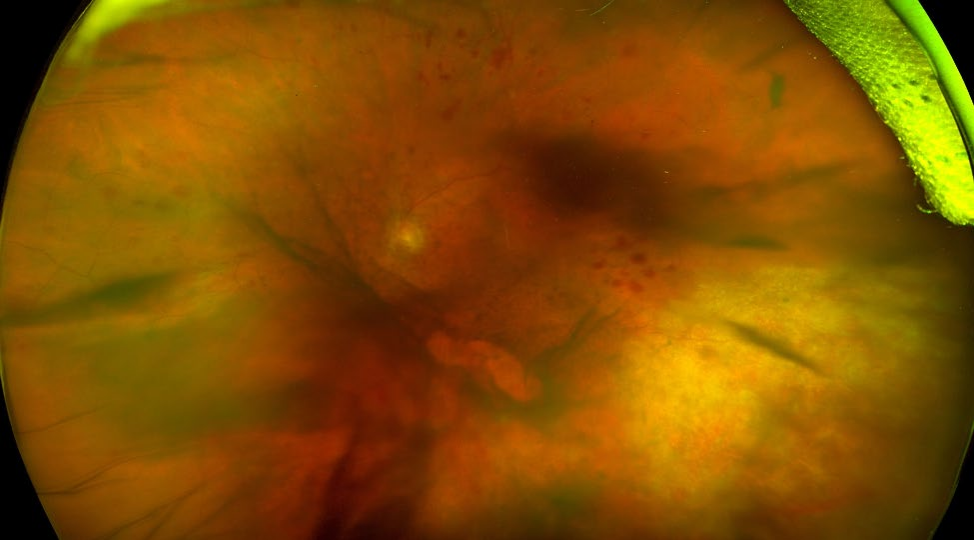
Wide-field fundus photograph of the left eye on initial presentation showing vitreous haze, diffuse retinal hemorrhages, and inferotemporal retinal whitening

**Fig. 2:**
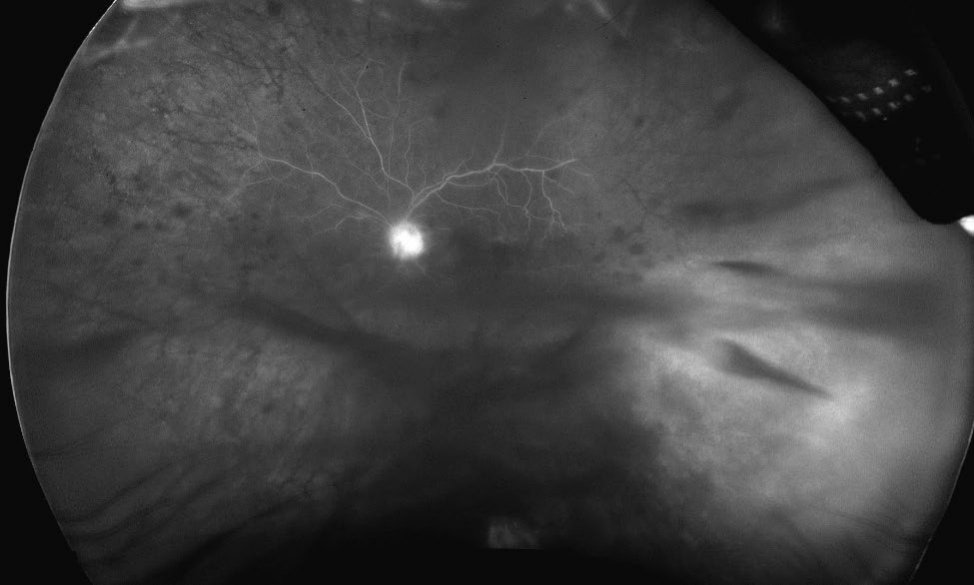
Wide-field late-phase fluorescein angiogram of the left eye on initial presentation showing optic nerve head leakage, cystoid macular edema, and severe retinal non-perfusion

An 80-year-old female presented with one month of floaters and blurry vision in the left eye. Her past ocular history was significant for age-related cataracts. Her past medical history was significant for chronic kidney disease along with seronegative rheumatoid arthritis that was being managed with intravenous abatacept 750 mg every 28 days, oral methotrexate 10 mg weekly, and oral prednisone 2 mg daily. On exam, her visual acuity was 20/20 in the asymptomatic right eye and 20/70 in the left eye. The right eye was quiet without significant retinal findings. The left eye was found to have 1 + flare in the anterior chamber and 2 + mixed white and red blood cells in the vitreous with grade 2 haze. Retinal exam of the left eye showed diffuse retinal hemorrhages with sclerotic-appearing vessels and an area of retinal whitening in the inferotemporal periphery (Fig. 1). Fluoroscein angiography displayed optic disk leakage, petaloid macular leakage, and significant peripheral non-perfusion (Fig. 2). Due to suspicion for infectious retinitis, an anterior chamber paracentesis of the left eye was performed with aqueous fluid sent for directed PCR for CMV, Herpes simplex virus (HSV) types 1 and 2, and Varicella zoster virus (VZV). The PCR testing returned positive for CMV with 996,000 copies per mL and negative HSV and VZV testing. Serum CMV PCR testing also returned positive with less than 1,000 copies per mL. Treponemal antibody testing was negative.

**Fig. 3:**
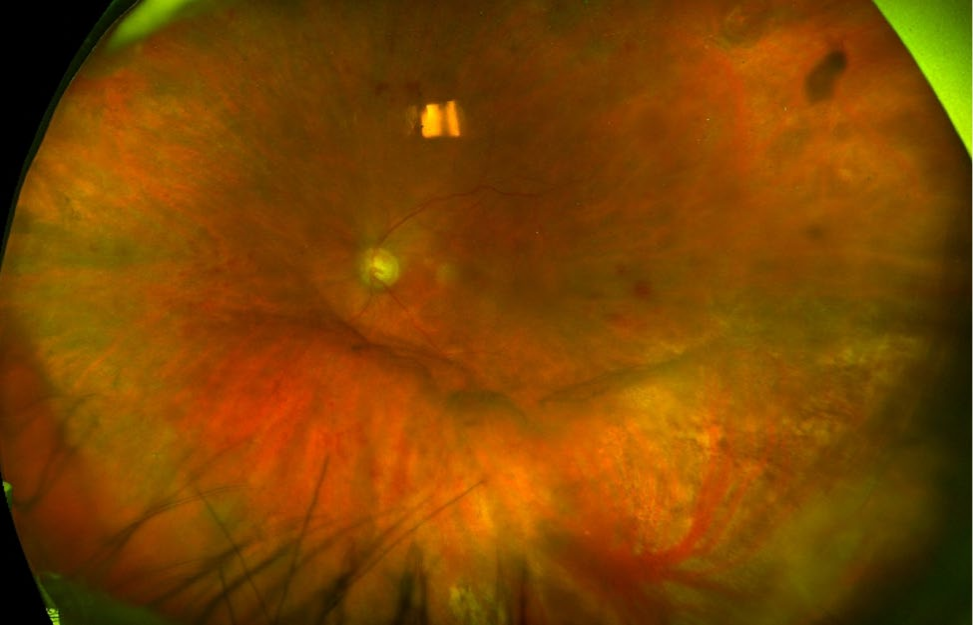
Wide-field fundus photograph of the left eye following initial treatment with intravitreal and oral ganciclovir, showing improved vitreous haze and retinal hemorrhages with resolved retinal whitening and resulting retinal atrophy in the inferotemporal periphery

A diagnosis of CMV CRN was made, and the patient was started on weekly intravitreal ganciclovir injections in the left eye along with oral valganciclovir through the infectious disease service at a dose of 450 mg daily based on her renal function. Her treating rheumatologist also discontinued methotrexate and started leflunomide 10 mg daily, which was also dose-adjusted for her renal function. Three intravitreal ganciclovir injections were given. The retinitis responded well to this regimen with resulting retinal atrophy in the area of prior retinitis (Fig. 3).

**Fig. 4:**
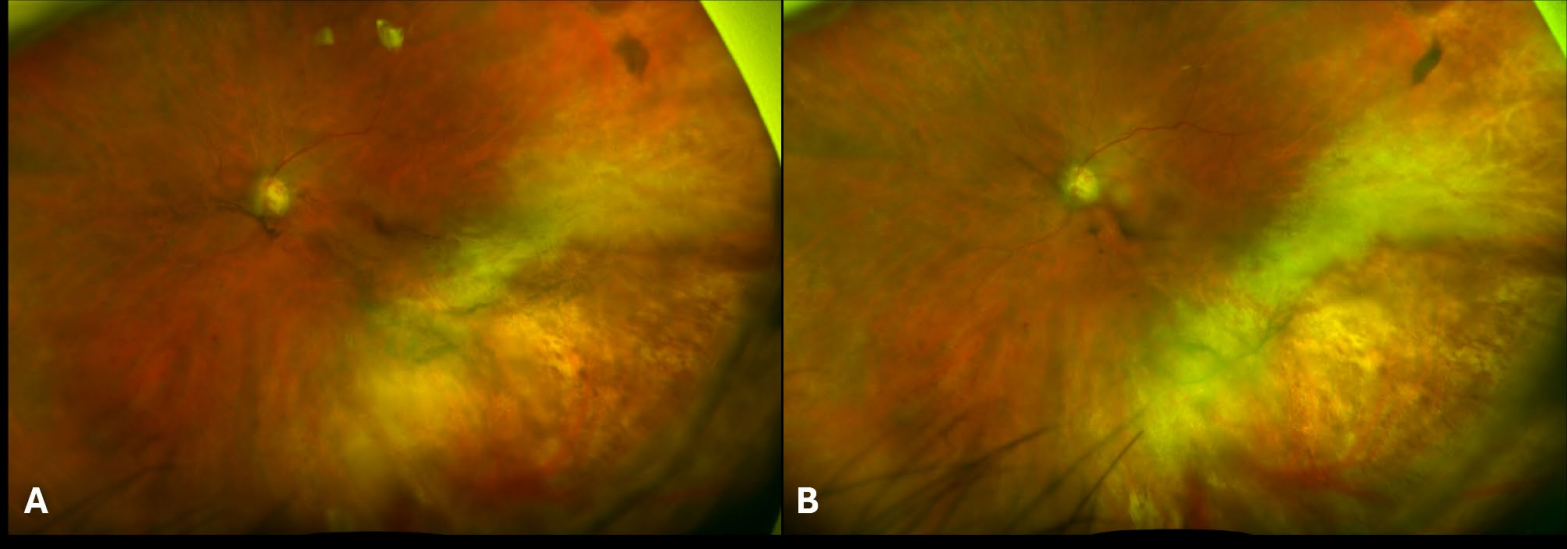
Wide-field fundus photograph of the left eye showing recurrence of retinal whitening (**4A**) with further progression (**4B**) despite intravitreal and oral ganciclovir

**Fig. 5:**
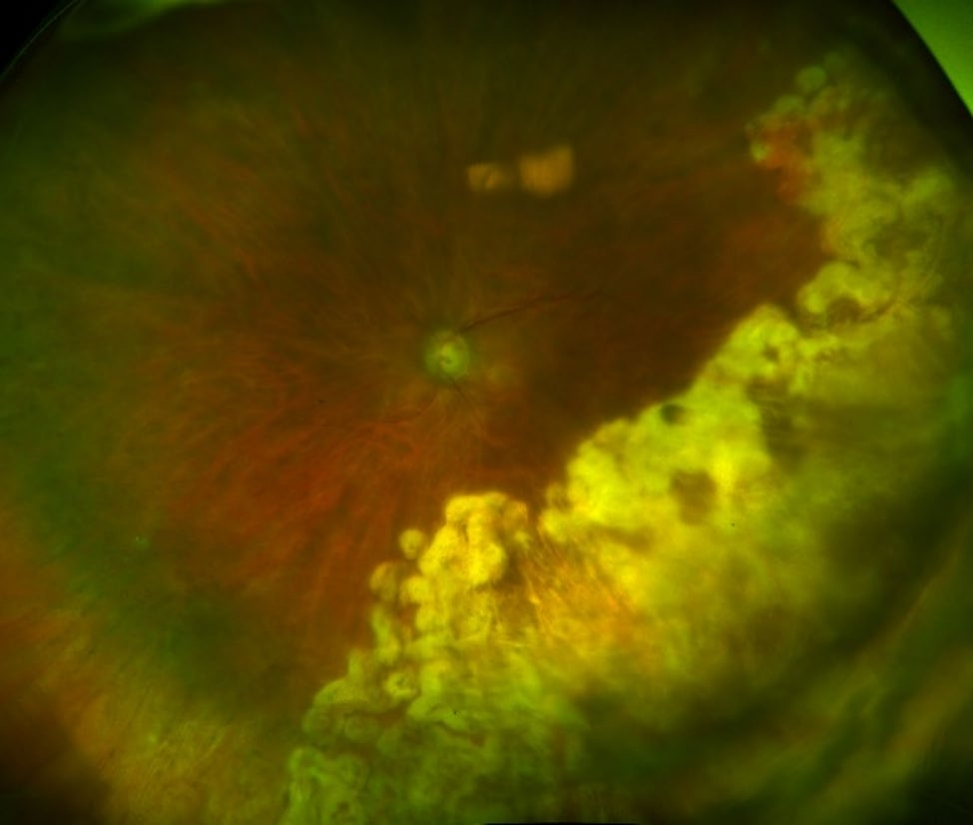
Wide-field fundus photograph of the left eye at final follow-up showing inactive retinitis with surrounding laser scars

Six months after initial presentation, cystoid macular edema (CME) developed in the left eye. The CME was unresponsive to topical corticosteroids so a sub-Tenon’s triamcinolone acetonide (STA) injection was given. Approximately one month following the STA, the patient discontinued leflunomide due to gastrointestinal symptoms and valganciclovir was also discontinued following six months of treatment. Four weeks following the injection, the CME had resolved, however re-activation of the retinitis was noted three months following the injection (Fig. 4A). The patient was restarted on intravitreal ganciclovir injections twice weekly to the left eye along with oral valganciclovir at induction dosing. Despite two weeks of this therapy, the retinitis continued to progress (Fig. 4B). Due to concern for ganciclovir resistance, the patient was switched to twice weekly intravitreal foscarnet injections and valganciclovir was switched to letermovir 480 mg daily. Rheumatology also discontinued abatacept and prednisone and restarted leflunomide at 20 mg daily. A total of 18 intravitreal foscarnet injections were given. This regimen successfully controlled the retinitis, although the course was complicated by a rhegmatogenous retinal detachment that was surgically repaired. The patient has now continued with letermovir 480 mg daily and leflunomide 20 mg daily for approximately 2.5 years without reactivation of the retinitis (Fig. 5) or need for further intravitreal anti-viral injections and with adequate control of her rheumatoid arthritis. The CME has been controlled with the use of topical corticosteroids only. Best-corrected visual acuity in the left eye at last follow-up was 20/50 and the right eye has remained uninvolved.

## Discussion

We present a case of CMV CRN with ganciclovir resistance that was successfully managed with letermovir and leflunomide.

Viral retinitis is a rare but vision-threatening disease. Acute retinal necrosis (ARN) is typically caused by HSV or VZV and classically presents in immunocompetent individuals. It is characterized by rapidly progressive retinitis with significant intraocular inflammation, the latter of which is thought to stem from an intact host immune response [15]. In contrast, CMV retinitis is seen in severely immunocompromised patients and is typified by slowly progressive retinal necrosis and minimal intraocular inflammation. The more recently described CMV CRN that is seen in “partially” immunocompromised people seems to share some features of both these entities: the chronic/subacute course of CMV retinitis with the associated intraocular inflammation and occlusive retinal vasculitis present in ARN [8]. 

Case reports of CMV CRN have described a variety of potential etiologies for the underlying mild immunocompromised state. These include: immunosenescence from aging, presence of diabetes mellitus, and the use of non-cytotoxic immunosuppressive agents, such as prednisone, anti-metabolites, and calcineurin inhibitors [8–14]. At this time, it is not clear if certain immunosuppressive agents may lead to a higher risk of CRN than others. Our patient was receiving treatment with abatacept, a biologic fusion protein that inhibits T-cell activation, along with low-dose methotrexate and prednisone at the time of CRN onset.

In the setting of immunosuppression for the treatment of a systemic autoimmune disease, initial treatment of CMV CRN could potentially consist of modification or discontinuation of these treatments to allow for immune restoration [12]. This is not always possible however, and systemic and/or intravitreal anti-viral treatments have regardless been utilized in the previously reported cases [8–14]. Ganciclovir, an inhibitor of CMV DNA polymerase, is commonly used as the first-line anti-viral agent in treating CMV infections. However, ganciclovir resistance develops in 40–50% of patients, particularly in the setting of chronic treatment, the need for multiple treatment courses, and inadequate absorption of oral ganciclovir. Ganciclovir resistance is typically due to mutations in the CMV phosphotransferase (UL97) or polymerase (UL54) genes [6, 16]. While our patient did not undergo CMV genetic analysis to confirm a resistance-conferring mutation, there was progression of retinitis despite two weeks of therapy, which, in the absence of genetic data, is considered refractory CMV [17]. 

With ganciclovir resistance, consideration can be given to switching to systemic foscarnet or cidofovir, however cross-resistance between ganciclovir and these two drugs is relatively high and both can be associated with significant side effects and toxicities [5, 6, 18]. Intravitreal anti-viral injections can be effective and avoid systemic toxicities. In our case, for example, multiple intravitreal foscarnet injections were successfully utilized to treat active retinitis that was not responding to intravitreal and oral ganciclovir. However, due to their short duration of action [19] and the subsequent need for frequent re-injection, intravitreal anti-viral injections are difficult to utilize chronically for prophylactic treatment.

Leflunomide, via its active metabolite teriflunomide, is an anti-metabolite that inhibits B- and T-cell replication and has been found to be effective in the treatment of inflammatory disease, particularly rheumatoid arthritis, for which it appears to have similar efficacy as methotrexate [20]. The anti-CMV properties of leflunomide arise from interference with viral capsid assembly, so there does not appear to be cross-resistance with the traditional anti-viral agents which inhibit CMV DNA replication [18, 21–23]. Prior studies have reported success with the use of leflunomide at doses of up to 100 mg daily in treating ganciclovir-resistant CMV viremia and CMV retinitis [18, 22–24]. GI side effects and transaminitis are the most common potential toxicities. In our case, leflunomide 20 mg daily was tolerated and appeared to provide benefit for both CRN and rheumatoid arthritis.

Letermovir is a newer anti-CMV medication that was approved in 2017 in the United States for the treatment of CMV infection following HSCT. Letermovir acts via inhibition of the CMV viral terminase complex (UL56), also a different mechanism of action than traditional anti-virals [25, 26]. It has been found to be effective in the treatment of CMV anterior uveitis and drug-resistant CMV retinitis with minimal side effects and without the need for routine laboratory monitoring [6, 16, 25, 26]. There is evidence from in-vitro studies that UL56 mutations conferring letermovir resistance can emerge quickly, although there are only few cases of this being found in clinical practice [6, 27]. 

The local immunosuppression induced by the STA injection, in conjunction with the patient discontinuing leflunomide and letermovir shortly after the injection, likely also contributed to the re-activation of retinitis. Clinicians should be cautious in utilizing local corticosteroid injections in the setting of CMV CRN, and it would seem prudent to continue systemic anti-viral prophylaxis if injections are utilized to treat secondary inflammation such as CME.

While a recently described disease, the incidence of CMV CRN may increase in the future as the use of non-cytotoxic immunosuppressive therapies that result in relatively mild immunosuppression also increases. Treatment with ganciclovir is effective but frequently leads to resistance, as in our case. In this situation, combination use of letermovir and leflunomide, particularly in the setting of rheumatoid arthritis where leflunomide can also have an anti-inflammatory effect, can be considered. The use of multiple anti-viral therapies with different mechanisms of action may also lead to decreased risk of resistance, as has been demonstrated in other chronic viral infections [28]. 

A limitation of this case report is the lack of CMV genetic analysis in our patient to identify a resistance-conferring mutation.

## Conclusions

To the best of our knowledge, this is the first reported case of ganciclovir-resistant CMV- CRN, which in this case was successfully managed with letermovir and leflunomide along with reduction of systemic immunosuppressive medications. Leflunomide was also effective in managing the patient’s rheumatoid arthritis. Treatment of these complex patients often requires coordination between multiple specialties, including ophthalmology, infectious disease, and rheumatology.

## Data Availability

No datasets were generated or analysed during the current study.

## Abbreviations

CMV: Cytomegalovirus
HIV: Human immunodeficiency virus
HSCT: Hematopoietic stem cell transplant
CRN: Chronic retinal necrosis
HSV: Herpes simplex virus
VZV: Varicella zoster virus
CME: Cystoid macular edema
STA: Sub-Tenon’s triamcinolone acetonide
ARN: Acute retinal necrosis
